# Phase II study of R–CVP followed by rituximab maintenance therapy for patients with advanced marginal zone lymphoma: consortium for improving survival of lymphoma (CISL) study

**DOI:** 10.1186/s40880-019-0403-7

**Published:** 2019-10-16

**Authors:** Sung Yong Oh, Won Seog Kim, Jin Seok Kim, Seok Jin Kim, Dok Hyun Yoon, Deok-Hwan Yang, Won Sik Lee, Hyo Jung Kim, Ho-Young Yhim, Seong Hyun Jeong, Jong Ho Won, Suee Lee, Jee Hyun Kong, Sung-Nam Lim, Jun Ho Ji, Kyung A. Kwon, Gyeong-Won Lee, Jae Hoon Lee, Ho Sup Lee, Ho-Jin Shin, Cheolwon Suh

**Affiliations:** 1 Department of Internal Medicine, Dong-A University Hospital, Busan, 49201 Republic of Korea; 2 Department of Medicine, Samsung Medical Center, Sungkyunkwan University School of Medicine, Seoul, 06351 Republic of Korea; 3 Division of Hematology, Department of Internal Medicine, Yonsei University College of Medicine, Seoul, 03722 Republic of Korea; 4 Department of Oncology, Asan Medical Center, University of Ulsan College of Medicine, Songpa-gu, Seoul, 05505 Republic of Korea; 5 Department of Hematology-Oncology, Chonnam National University Hwasun Hospital, Hwasun, 58128 Republic of Korea; 6 Department of Hematology, Busan Paik Hospital, Inje University College of Medicine, Busan, 04511 Republic of Korea; 7 Department of Internal Medicine, Hallym University Sacred Heart Hospital, Hallym University College of Medicine, Anyang, 14068 Republic of Korea; 8 Department of Internal Medicine, Chonbuk National University Hospital, Jeonju, 54907 Republic of Korea; 9 Department of Hematology-Oncology, Ajou University School of Medicine, Suwon, 16499 Republic of Korea; 10 Department of Internal Medicine, Soonchunhyang University Seoul Hospital, Soonchunhyang University College of Medicine, Seoul, 04401 Republic of Korea; 11 Division of Hematology-Oncology, Department of Internal Medicine, Wonju Severance Christian Hospital, Yonsei University College of Medicine, Wonju, 26426 Republic of Korea; 12 Department of Internal Medicine, Haeundae Paik Hospital, Inje University College of Medicine, Busan, 48108 Republic of Korea; 13 Division of Hematology and Oncology, Department of Internal Medicine, Samsung Changwon Hospital, Sungkyunkwan University School of Medicine, Changwon, 51353 Republic of Korea; 14 Division of Hematology-Oncology, Department of Internal Medicine, Dongnam Institute of Radiological and Medical Sciences, Busan, 46033 Republic of Korea; 15 Division of Hematology-Oncology, Department of Internal Medicine, Gyeongsang National University Hospital, Gyeongsang National University College of Medicine, Jinju, 52727 Republic of Korea; 16 Department of Internal Medicine, Gachon University Gil Medical Center, Incheon, 21565 Republic of Korea; 17 Department of Internal Medicine, Kosin University Gospel Hospital, Kosin University College of Medicine, Busan, 49267 Republic of Korea; 18 Division of Oncology, Department of Internal Medicine, Pusan National University Hospital, Busan, 49241 Republic of Korea

**Keywords:** Marginal zone, Lymphoma, Advanced stage, Rituximab, Cyclophosphamide, Vincristine, Maintenance, Multicenter, Open label, Survival

## Abstract

**Background:**

The response rate and survival improvement for rituximab, a CD20-targeting monoclonal antibody, have been demonstrated in marginal zone lymphoma (MZL) as monotherapy and in combination with chemotherapeutic regimens, yet relapses still occur despite treatment completion. Thus, extending the period of remission in MZL patients remains an essential goal. This multicenter, single-arm, open-label phase II study evaluated the survival efficacy of 2 years of rituximab-maintenance therapy in patients with stage III–IV CD20-positive MZL who had responded to first-line R–CVP (rituximab, cyclophosphamide, vincristine, and prednisolone). The objective of this study was to determine whether rituximab maintenance following R–CVP warrants further investigation.

**Methods:**

Prior to rituximab-maintenance therapy, patients received 6–8 cycles of first-line R–CVP therapy for stage III–IV MZL. Rituximab (375 mg/m^2^), cyclophosphamide (750 mg/m^2^), and vincristine (1.4 mg/m^2^; maximum 2 mg) were administered via an intravenous infusion on day 1 of each 3-week cycle, while oral prednisolone (100 mg) was given on days 1–5 of each 3-week cycle. The patients who achieved complete response (CR), partial response (PR), or stable disease (SD) to R–CVP treatment, were prescribed rituximab-maintenance therapy which was administered intravenously at a dose of 375 mg/m^2^ every 8 weeks for up to 12 cycles. The primary endpoint was progression-free survival (PFS). Secondary endpoints were overall survival (OS) and treatment safety.

**Results:**

47 patients were enrolled, of whom, 45 (96%) received rituximab-maintenance treatment. Fifteen (33%) patients had nodal MZL. Following R–CVP first-line therapy, 20 (44%), 22 (49%), and 3 (7%) patients achieved CR, PR, and SD, respectively. After a median follow-up of 38.2 months, their observed 3-year PFS rate was 81%. During the rituximab-maintenance, 6 PR and 1 SD patients achieved CR following the administration of R–CVP. Elevated LDH and the presence of B symptoms were found to be significant prognostic factors for PFS (*P *= 0.003) and demonstrated a 3-year OS rate of 90%. Rituximab-maintenance therapy was well tolerated, and the common treatment-emergent adverse events were sensory neuropathy (18%), myalgia (13%), fatigue (9%), and neutropenia (9%).

**Conclusion:**

Rituximab-maintenance therapy following first-line R–CVP demonstrated good PFS in patients with stage III–IV MZL, in addition to a favorable toxicity profile.

*Trial registration* clinicaltrials.gov: NCT01213095

## Background

Marginal zone lymphoma (MZL) is a B cell non-Hodgkin’s lymphoma (NHL) that accounts for approximately 5%–17% of all NHL in adults [1]. In Korea, MZL is the second most frequent histological NHL subtype after diffuse large B-cell lymphoma, constituting one-fifth of all NHL cases [2]. The three major subtypes of MZL defined by the World Health Organization (WHO)—are splenic MZL, mucosa-associated lymphoid tissue (MALT) MZL, and nodal MZL [3, 4], which are determined by the anatomical location of disease-initiating B-cells [5]. MALT is the most common MZL subtype, with an estimated 5-year overall survival (OS) and progression-free survival (PFS) > 90% and 70% while nodal MZL has been associated with most frequent relapse cases [2, 6–8]. Overall, MZL is characterized by an indolent clinical course [5], yet remission is often followed by multiple relapses [9, 10], highlighting the need for tolerable maintenance treatments that can extend the remission periods induced by first-line therapies.

In B-cell malignancies, rituximab (Mabthera^®^; Roche, Basel, Switzerland) was the first targeted therapy drug which caused a paradigm shift in disease treatment [11]. Rituximab is a chimeric, monoclonal antibody targeting CD20, a cell surface antigen expressed during most stages of B-cell development [12], and is found on 95% of B-cell lymphoma cells [13]. The clinical efficacy of rituximab was first demonstrated in follicular lymphoma (FL) [14, 15] and it has since been prescribed for other subtypes of NHL, including MZL, with promising results [16–24]. In this study, we evaluated rituximab as a candidate maintenance therapy in patients with advanced MZL.

In MZL, the clinical activity of rituximab as a single agent therapy has been studied in a small, retrospective case study and phase II studies, which have demonstrated improved safety and outcomes with rituximab-monotherapy [16–18]. However, in a phase II study conducted by the International Extranodal Lymphoma Study Group (IELSG), patients with MALT lymphomas did not respond to treatment within 14–22 months [18]. However, numerous studies have demonstrated the efficacy and tolerability of rituximab in combination with chemotherapy regimens, with an overall improved response rate of up to 90%–100% [19–24]. A phase III study of rituximab in combination with chlorambucil (R–Cb) comparing the efficacy of R or Cb monotherapies against R–Cb combination treatment showed that the group receiving combination treatment had superior 5-year event-free survival (68% with R–Cb vs. 50% with R vs. 51% with Cb, *P* < 0.001) [25]. For patients who have received first-line R–CVP (rituximab, cyclophosphamide, vincristine, and prednisolone) combination immunochemotherapy, the efficacy and safety of the regimen has been demonstrated in patients with untreated stage III–IV MZL, in the context of a phase II study conducted by the Consortium for Improving Survival of Lymphoma (CISL) [24]. The overall response rate achieved with R–CVP in the previous CISL study was 88%, with 60% of patients achieving complete response (CR) [24]. The 3-year PFS and OS rates were 59% and 95%, respectively [24]. In another phase II trial, R–B (rituximab–bendamustine) treatment for patients with MALT lymphoma, also reported a 100% CR and 87.7% event-free survival rate at 7 years after treatment (95% CI 76.0–94.0) [26, 27].

Despite the improved response and progression or event-free survival rates achieved with first-line rituximab-containing regimens, relapses still persist once the treatment is completed [16–24]. Typically, patients suffer multiple relapses, and subsequent lines of therapy for MZL patients achieve progressively shorter responses [9], therefore, extending the period of remission for MZL patients remains an essential goal. One potential strategy is the use of maintenance therapy once a response has been achieved with first-line therapy. Several NHL studies have previously evaluated rituximab as a maintenance agent [14, 15, 28–31]. FL patients who receive rituximab-maintenance therapy after responding to first-line rituximab and chemotherapy experience significantly longer PFS compared to those receiving standard treatment [14, 15]. Similar results were reported for the randomized phase III primary rituximab and maintenance (PRIMA) trial, in which 2 years of rituximab-maintenance therapy significantly improved the 6-year PFS in FL patients who responded to first-line rituximab and chemotherapy combination treatment, compared to patients on standard therapy [R–CVP or R–CHOP (rituximab, cyclophosphamide, doxorubicin, vincristine, and prednisolone)] alone [28, 29].

The studies described above indicate that adding rituximab-maintenance therapy to a rituximab-based immunochemotherapeutic regimen may be an effective strategy for extending remission in patients with advanced MZL. Here we report the results of a phase II study evaluating the effect of rituximab-maintenance treatment following R–CVP as a first-line therapy on the survival of MZL patients.

## Materials and methods

### Study design and participants

This was a multicenter, open-label, non-comparative phase II study conducted in medical centers across South Korea (ClinicalTrials.gov identifier NCT01213095). Patients aged ≥ 20 years with histologically confirmed, Ann Arbor stage III–IV, CD20-positive MZL were eligible for this study. The patients who had achieved CR, partial response (PR), or stable disease (SD) after 6 or 8 cycles of first-line R–CVP combination therapy, as defined by the revised International Working Group (IWG) response criteria for malignant lymphoma were enrolled to the R-maintenance clinical trial [32].

Enrolment was possible even without symptom for first-line treatment with R–CVP. Other eligibility criteria were presence of at least one bi-dimensionally measurable lesion (≥ 2 cm by conventional computed tomography [CT], ≥ 1 cm by spiral CT, ≥ 2 cm skin lesion, or ≥ 2 cm on physical examination) at first line R–CVP treatment; had an Eastern Clinical Oncology Group (ECOG) performance status (PS) score ≤ 2; and adequate renal, liver and bone marrow (BM) functions at baseline of R-maintenance enrollment.

Patients who had received prior chemotherapy or radiotherapy for MZL were excluded from the study, as were those with a large cell component > 10%, central nervous system involvement, or previous malignancy in the past 5 years with the exceptions of curatively treated non-melanoma skin cancer, in situ carcinoma of the cervix uteri, or thyroid cancer with completed active treatment and no evidence of recurrence over a period of 1 year. Informed consent was obtained from all patients for their participation in the study. Informed consent and patients’ enrolment were acquired after the first line R–CVP treatment. Investigators clearly discussed with the patients for their treatment option including “watchful wait”. This clinical trial obtained informed consents for R-maintenance treatment. This study was conducted in accordance with the Declaration of Helsinki and International Conference on Harmonisation Good Clinical Practice guidelines. The study design was reviewed and approved by the relevant independent ethics committees for each investigational site. All authors had access to primary clinical trial data.

### Procedures

Prior to rituximab-maintenance therapy, patients received 6–8 cycles of first-line R–CVP therapy. Rituximab (Roche Pharm Co., Ltd., Basel, Switzerland) (375 mg/m^2^), cyclophosphamide (Bukwang Pharm Co., Ltd, Seoul, Korea) (750 mg/m^2^), and vincristine (1.4 mg/m^2^; maximum 2 mg) were administered via an intravenous infusion on day 1 of each 3-week cycle, while oral prednisolone (Yuhan Corporation Co., Ltd, Seoul, Korea) (100 mg) was given on days 1–5 of each 3-week cycle. Screening assessments of tumor response before initiating rituximab-maintenance were performed on day 21 of the final R–CVP cycle, and within the 14 days prior to the first dose of rituximab-maintenance treatment.

Rituximab-maintenance therapy was administered intravenously at a dose of 375 mg/m^2^ every 8 weeks for up to 12 cycles. To enhance infusion safety, infusion rates and premedication were administered according to the rituximab’s prescribing information [33]. Tumor responses were assessed according to the IWG criteria [32] at screening and following every two cycles of rituximab-maintenance therapy, or when disease progression was suspected. Evaluation of response to therapy included physical examination, serum lactate dehydrogenase (LDH), CT or magnetic resonance imaging of initially involved sites, and positron emission tomography (PET) or PET-CT. In cases of initial BM involvement, bilateral BM aspiration and biopsies were performed after chemotherapy completion to confirm CR.

Adverse events (AEs) were graded according to the National Cancer Institute Common Terminology Criteria for Adverse Events (NCI-CTCAE, Version 4.03). Safety monitoring continued up to 30 days after the final cycle of rituximab-maintenance. Follow-up was continued for 3 years after completing the study treatment, and the tumors were assessed in imaging studies performed every 6 months until disease progression was detected, at which point information on survival and new lymphoma treatment were updated every 6 months until death.

### Outcomes

The primary endpoint was 3-year PFS, defined the length of time during and after the R–CVP treatment of MZL that a patient lives with the disease but it does not get worse. Secondary endpoints were (1) overall survival (OS), defined from the length of time of R–CVP treatment commencement until death due to any cause or the date of the last follow-up, and (2) treatment safety.

### Statistical analyses

This trial was designed according to the Simon “optimal” design for phase II trials and aimed to determine whether rituximab-maintenance following R–CVP could improve PFS [33]. Based on literature analyses [7, 24], the baseline 3-year PFS rate was expected to be 50%, with an anticipated treatment difference of 20%. Assuming a drop-out rate of 10%, a total of 47 patients were required to achieve a power of 80% to detect a 20% treatment difference with an alpha of 0.05. PFS was defined as the time R–CVP treatment started to the first recorded incidence of relapse, disease progression, death due to any cause, or last date of follow up for the enrolled patients who did not progress.

The intent-to-treat population (for efficacy analysis) and safety population (for safety analysis) both included enrolled patients who received at least one dose of rituximab-maintenance therapy. Time-to-event data were estimated using the Kaplan–Meier method. The Cox proportional hazards model was used to estimate the hazard ratio (HR) and the corresponding 95% CI with regard to the low-risk group. All reported *P* values were two-sided, and a *P* value < 0.05 was considered significant. All analyses were conducted using the Statistical Package for Social Sciences version 20.0 for Windows (SPSS Inc., Chicago, IL, USA).

## Results

**Fig. 1:**
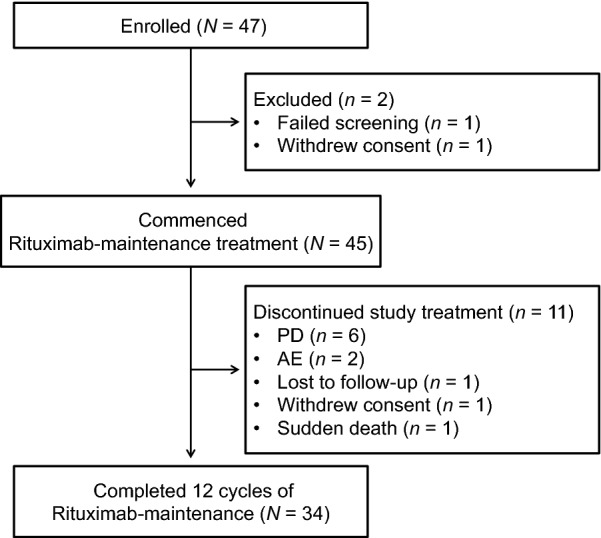
Patient disposition. Flow chart showing the number of patients who were enrolled, commenced rituximab-maintenance treatment, and completed the rituximab-maintenance treatment. *AE* adverse event, *PD* progressive disease

A total of 47 patients were enrolled into this trial from a total of 18 centers, of whom, 45 (96%) received rituximab-maintenance treatment. One (2%) patient failed screening due to thyroid cancer, and one (2%) patient withdrew consent (Fig. 1). The first patient of the trial was enrolled on October 19, 2010, and the date of last follow-up was on February 4, 2016. In total, 34 (72%) patients completed the planned 12 cycles of rituximab-maintenance therapy (Fig. 1). Six (13%) patients discontinued due to progressive disease (PD), while two (4%) discontinued due to AEs, one (2%) was lost to follow-up, one (2%) withdrew consent, and one (2%) died (pneumonia, after 11 cycles) prior to the rituximab-maintenance treatment completion.

Baseline patient demographics and disease characteristics are summarized in Table 1. The median age was 54 years (range, 33–77 years), and 43 (96%) patients had an ECOG performance score ≤ 1. In total, 15 (33%) patients had nodal MZL and 30 (67%) had MALT MZL. Following R–CVP first-line therapy, 20 (44%), 22 (49%), and 3 (7%) patients achieved CR, PR, and SD, respectively (Table 1). The number of patients who received 6 or 8 cycles of prior R–CVP therapy were 10 (22%) and 35 (78%), respectively (Table 1).

**Table 1:** Baseline demographics and disease characteristics in the intent-to-treat population

Characteristics	Number of cases (%)
Age	
Median, years (range)	54 (33–77)
< 60	29 (64.4)
≥ 60	16 (35.6)
Sex	
Male	32 (71)
Female	13 (29)
ECOG performance score	
0–1	43 (96)
2	2 (4)
Ann Arbor stage at diagnosis	
III	11 (24)
IV	34 (76)
LDH	
Within normal range	35 (78)
Elevated	7 (16)
Unchecked	3 (7)
B symptoms^a^	
Absent	38 (84)
Present	7 (16)
BM involvement	
Absent	34 (76)
Present	11 (24)
Histology	
Nodal MZL	15 (33)
MZL of MALT-type	30 (67)
Lung	8 (18)
Ocular and adnexa	6 (13)
Stomach	4 (9)
Bone	2 (4)
Nasopharynx	2 (4)
Multiple MALT sites	3 (7)
Others^b^	5 (11)
IPI score	
1	13 (29)
2	21 (47)
3	9 (20)
4	2 (4)
Response to prior R–CVP	
CR	20 (44)
PR	22 (49)
SD	3 (7)
No. first line R–CVP (6 cycles)	10 (22)
Treatment cycles (8 cycles)	35 (78)

Values are expressed as n (%) unless indicated otherwise

*BM* bone marrow, *CR* complete response, *ECOG* Eastern Clinical Oncology Group, *IPI* International Prognostic Index, *LDH* lactate dehydrogenase, *MALT* mucosa-associated lymphoid tissue, *MZL* marginal zone B-cell lymphoma, *PR* partial response, *R*–*CVP* rituximab cyclophosphamide vincristine prednisolone, *SD* stable disease

^a^Fever, night sweats, and/or weight loss

^b^One case each in the kidney, liver, nasal cavity, subcutaneous tissue, and small intestine

**Fig. 2:**
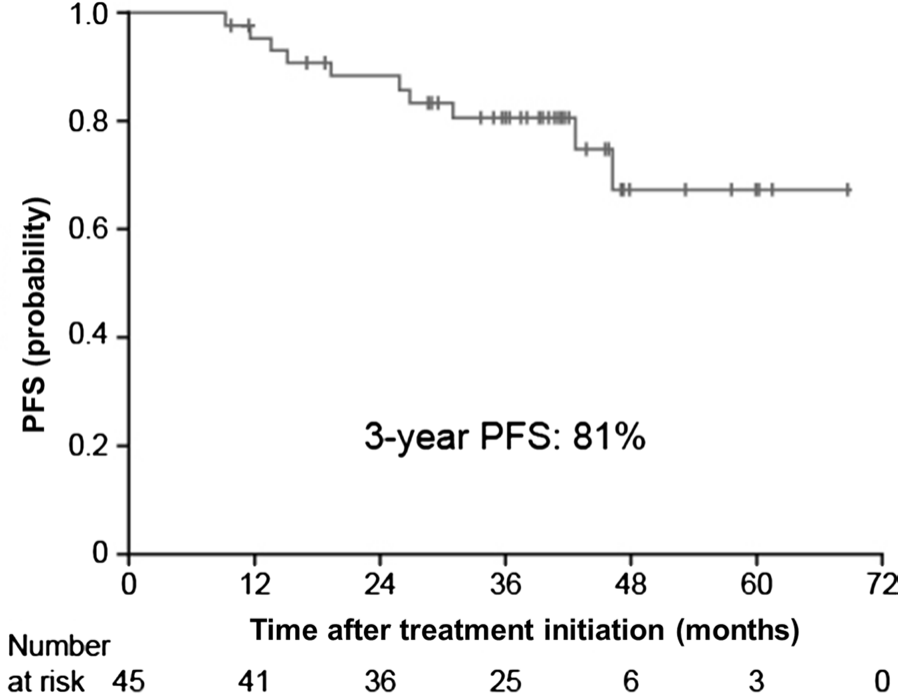
PFS following R–CVP and rituximab-maintenance therapy in the intent-to-treat population. Kaplan–Meier plot of PFS in patients with advanced MZL treated with rituximab-maintenance following first-line R–CVP therapy. *MZL* marginal zone lymphoma, *PFS* progression-free survival, *R*–*CVP* rituximab cyclophosphamide vincristine prednisolone

**Fig. 3:**
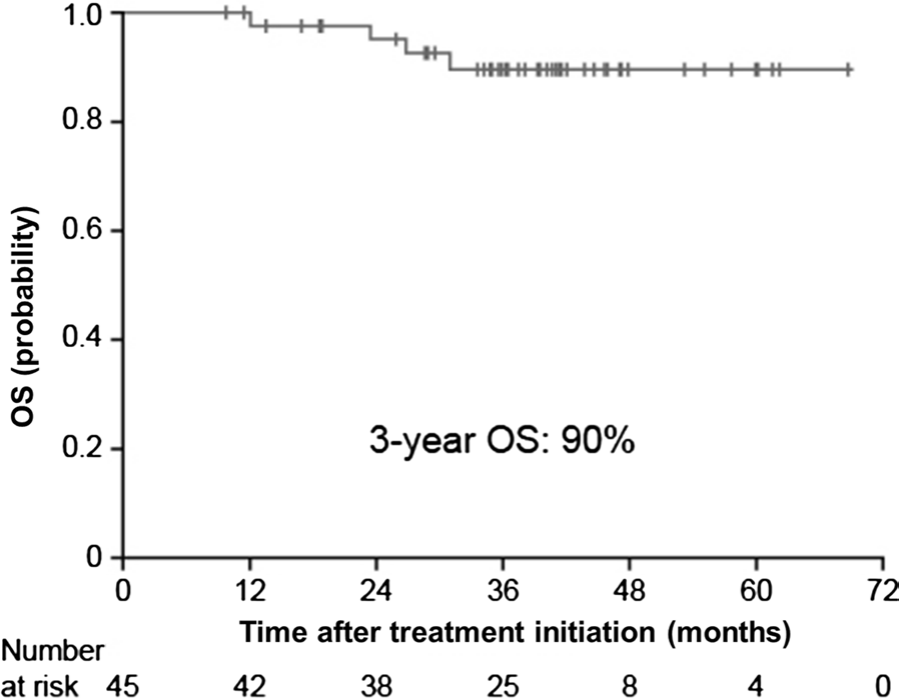
OS following R–CVP and rituximab-maintenance therapy in the intent-to-treat population. Kaplan–Meier plot of OS for patients with advanced MZL treated with rituximab-maintenance following first-line R–CVP therapy. *MZL* marginal zone lymphoma, *OS* overall survival, *R*–*CVP* rituximab cyclophosphamide vincristine prednisolone

After a median follow-up of 38.2 months, the 3-year PFS rate was found to be 81% (Fig. 2). During the rituximab-maintenance therapy, 6 PR patients and 1 SD patient achieved CR following R–CVP. Univariate analyses showed that elevated LDH (HR 6.819; 95% CI 1.885–24.667; *P *= 0.003) and the presence of B symptoms (HR 0.130; 95% CI 0.034–0.500; *P *= 0.003) to be significant prognostic factors for PFS following rituximab-maintenance (Table 2). MZL subtype was not a significant prognostic factor for PFS, nor was response to R–CVP (CR vs. < CR; Table 2). After a median follow-up of 38.2 months, the 3-year OS rate was found to be 90% (Fig. 3).

**Table 2:** Univariate analyses of prognostic factors for PFS in the intent-to-treat population

Variable	*N*/*n*	PFS		
		HR	95% CI	*P* value
Gender (male vs. female)	32/13	0.025	0.000–6.051	0.187
Age (< 60 years vs. ≥ 60 years)	29/16	2.663	0.743–9.540	0.132
ECOG performance status (0–1 vs. 2–3)	43/2	4.756	0.582–38.870	0.146
Ann Arbor stage (III vs. IV)	11/34	31.820	0.072–13993.516	0.265
Elevated LDH (no vs. yes)	38/7	6.819	1.885–24.667	0.003
BM involvement (absent vs. present)	34/11	3.313	0.862–12.736	0.081
B symptoms^a^ (present vs. absent)	7/38	0.130	0.034–0.500	0.003
IPI score (1 vs. 2–4)	13/32	4.951	0.622–39.401	0.131
Extranodal MZL (present vs. absent)	30/15	0.570	0.157–2.062	0.391
R–CVP response (CR vs. < CR)	20/25	0.388	0.097–1.544	0.179

*BM* bone marrow, *CR* complete response, *CI* confidence interval, *ECOG* Eastern Clinical Oncology Group, *HR* hazard ratio, *IPI* International Prognostic Index, *LDH* lactate dehydrogenase, *MZL* marginal zone B-cell lymphoma, *PFS* progression-free survival, *R–CVP* rituximab cyclophosphamide vincristine prednisolone

^a^Fever, night sweats, and/or weight loss

A total of 51 treatment-emergent AEs (TEAEs) were reported during the study, the majority of which were grade 1 or 2 (Table 3). Of the two patients who discontinued the treatment due to AEs, one experienced abdominal pain and the other had recurrent pneumonia. In total, four deaths occurred during the study (one sepsis, one PD, and two pneumonia), one (pneumonia) of which was related to the treatment. TEAEs experienced by more than one patient are summarized in Table 3. The most frequent treatment-related TEAEs were sensory neuropathy (18%), myalgia (13%), fatigue (9%), and neutropenia (9%). All cases of sensory neuropathy and myalgia were of grade 1 or 2. Of the four cases who experienced fatigue, two were of grade 1 and two were of grade 3, while three of the four cases of neutropenia were classified as grade 3–4.

**Table 3:** Summary of TEAEs (safety population)

TEAEs	Number of cases (%)
Total number of TEAEs	51
TEAEs	
Grade 1	23 (51)
Grade 2	17 (38)
Grade 3	5 (11)
Grade 4	6 (13)
TEAEs leading to treatment discontinuation	2
Deaths	4
Treatment-related	1
Treatment-related TEAEs reported in > 1 patient	
Sensory neuropathy	8 (18)
Myalgia	6 (13)
Fatigue	4 (9)
Neutropenia	4 (9)
Anorexia	2 (4)
General weakness	2 (4)
Headache	2 (4)
Insomnia	2 (4)
Pneumonia	2 (4)
Sepsis	2 (4)
Tinnitus	2 (4)
Urticaria	2 (4)

Values are expressed as n (%)

*TEAE* treatment-emergent adverse event

## Discussion

MZL, despite being heterogeneous malignancy and mostly indolent, its disease characteristics, clinical picture, and treatment algorithms vary considerably based on the subtype and site of involvement. Relapses are frequent, and subsequent lines of therapy achieve incrementally shorter responses [9, 10]. Therefore, extending the period of remission induced by first-line therapies is an essential goal in the treatment of MZL. The current multicenter study evaluated the efficacy and safety of 2 years of rituximab-maintenance therapy in stage III–IV MZL patients who had previously been treated with 6 or 8 cycles of R–CVP combination therapy. Here, rituximab-maintenance following first-line R–CVP therapy led to 3-year PFS and OS survival rates of 81% and 90%, respectively. Univariate analyses identified elevated LDH and the presence of B symptoms as significant prognostic factors for PFS. Rituximab-maintenance treatment following rituximab-based immunochemotherapy was generally tolerable in this study, indicating that rituximab-maintenance treatment is a viable option for MZL patients. Except for the 4 cases of neutropenia above of grade 3, other non-hematologic toxicities were mild. In addition, we hypothesized that several of the presented symptoms—sensory neuropathy, fatigue, and myalgia—could have been originated prior to R–CVP immunochemotherapy induction.

Historically, given the typically slow progression and poor curability of MZL, patients with advanced disease have been subjected to a “watch-and-wait” approach, whereby treatment is delayed until the patients’ disease progress or becomes symptomatic [30]. Numerous studies have asked the question of whether treatment with rituximab may be a more suitable approach for these patients. Indeed, rituximab single-agent therapy has been shown to provide clinical benefit in NHL, including MZL, which can be improved upon combination with chemotherapeutic regimens—fludarabine, chlorambucil, CVP, and bendamustine, which has been summarized as shown in Table 4 [19, 23–27].

**Table 4:** First-line immunochemotherapy for marginal zone lymphoma

Study	Regimen	Disease type	Trial phase type	No. of patients	Overall RR (CR + PR)	PFS	OS
Salar et al. [19]	R-fludarabine	Any stage MALT lymphoma	II	22	100% (62 + 38)	2-year, 88%	2-year, 100%
Zucca et al. [23, 25]	R–Cb	Any stage MALT lymphoma	III (R–Cb vs R vs Cb)	132 (total 401)	94.7% (78.8 + 15.9)	5-year, 68%^a^	5-year, 90%
Kang et al. [24]	R–CVP	Stage III/IV MZL	II	41	87.5% (60 + 27.5)	3-year, 59%	3-year, 94%
Salar et al. [26, 27]	R–B	Any stage MALT lymphoma	II	57	100%	7-year, 92.8%	7-year, 94.7%
Oh (present study)	R–CVP followed by R-maintenance	Stage III/IV MZL	II	45	–	3-year, 81%	3-year, 90%

*PFS* progression-free survival, *OS* overall survival, *CR* complete response, *PR* partial response, *MZL* marginal zone B-cell lymphoma, *MALT* mucosa-associated lymphoid tissue, *R–CVP* rituximab-cyclophosphamide, vincristine, and prednisolone, *R–Cb* rituximab–chlorambucil, *R–B* rituximab–bendamustine

^a^Event-free survival

The efficacy and safety of a first-line R–CVP immunochemotherapy regimen were demonstrated in patients with previously untreated stage III–IV MZL in a phase II study conducted by CISL [24]. In this previous CISL study, following 6–8 three-weekly cycles of R–CVP, the 3-year PFS and OS rates were 59% and 95%, respectively. In comparison to the present study, an equivalent R–CVP regimen followed by rituximab-maintenance therapy resulted in a 3-year PFS rate over 20% higher (81% vs. 59%) than the CISL study. The OS of the 2 studies did not differ substantially (previous vs. current CISL studies, 95% vs. 90%, respectively), which may have been as a result of the short follow-up duration and small sample size of the present study.

There is growing evidence of improved outcomes among NHL populations after rituximab-maintenance therapy (Table 5). A randomized phase II study comparing rituximab-maintenance or retreatment in 114 NHL patients who had previously been treated with chemotherapy reported significantly longer PFS in the rituximab-maintenance group comparing observation group until progression (31.3 vs. 7.4 months) [31]. Rituximab-maintenance therapy has also been evaluated following induction with rituximab-monotherapy in patients with stage III–IV small lymphocytic lymphoma and MZL patients in the randomized phase III rituximab extended schedule or retreatment (RESORT) trial [30]. In patients who responded to rituximab-induction, the median time for treatment failure was significantly improved from 1.4 years with rituximab retreatment at disease progression to 4.8 years with rituximab-maintenance [30]. Furthermore, rituximab-maintenance significantly improved the PFS of FL patients following rituximab-based first-line immunochemotherapy [14, 15, 28, 29].

**Table 5:** Rituximab maintenance therapy for indolent lymphoma

Study	Disease, treatment	Induction treatment	Maintenance schedule	Trial design	No. of patients	Outcomes	Study arm	Control arm	*P* value
Hainsworth et al. [31]	FL/SLL	R weekly (4 times)	4 weeks R q 6 months × 4 times	Randomized phase II	114	PFS (median)	31.3 months	7.4 months	0.007
Williams et al. [30]	SLL/MZL	R weekly (4 times)	R q 3 months till PD	Phase III	128	TTP (median)	4.8 years	1.4 years	0.012
Taverna et al. [37]	FL (including relapse)	R weekly (4 times)	R q 2 months for 8 month vs 5 years	Phase III	165	EFS	3.4 years (8 months)	5.3 year (5 years)	0.14
Salles et al. [28]	FL	R–CVP/R–CHOP/R-FCM	R q 2 months for 2 years	Phase III	1019	PFS (3 years)	74.9%	57.6%	< 0.0001
Rummel et al. [35]	MZL	R–B + 2R	R q 2 months for 2 years	Randomized phase II	104	PFS (median)	Not reached	92.2 months	0.008
Oh (present study)	MZL	R–CVP	R q 2 months for 2 years	Phase II	45	PFS (3 years)	81%	–	–

*PFS* progression-free survival, *EFS* event-free survival, *TTP* time to progression, *PD* progression of disease, *MZL* marginal zone B-cell lymphoma, *FL* follicular lymphoma, *SLL* small lymphocytic lymphoma, *R* rituximab, *MALT* mucosa-associated lymphoid tissue, *R–CVP* rituximab-cyclophosphamide, vincristine, and prednisolone, *R–CHOP* rituximab–cyclophosphamide, doxorubicin, vincristine, and prednisolone, *R–FCM* rituximab–fludarabine, cyclophosphamide, and mitoxantrone, *R–Cb* rituximab–chlorambucil, *R–B* rituximab–bendamustine

Although the current study did not contain a reference arm, sustained rituximab treatment is likely to have resulted in an overall higher total dose of rituximab in patients receiving maintenance treatment. Indeed, the investigators in the phase III RESORT study estimated a three-fold higher rituximab dose in their maintenance group compared to patients receiving retreatment only [30]. The lack of a direct comparator in this study means statistical analyses cannot be performed to evaluate the effects of adding rituximab-maintenance to R–CVP first-line therapy at this stage. Furthermore, only one rituximab-maintenance duration was evaluated in this study, which has been shown to impact efficacy. The randomized phase III SAKK 35/03 study, which compared short-term (8 months) and long-term (up to 5 years) administration of bi-monthly rituximab-maintenance following rituximab-monotherapy in patients with FL, found that long-term maintenance therapy increased toxicity without improving the event-free survival or OS [34]. Additional randomized controlled trials are required to fully evaluate the role of R maintenance. In the MAINTAIN trial, patients having induction therapy were treated with up to 6 cycles of bendamustine plus rituximab (B–R) plus two additional R cycles. Only patients responding to B–R were then randomized to either R maintenance (q 2 months for 2 years) or observation. The PFS was superior for 2 years of R maintenance therapy, with the median not yet reached vs. 92.2 months for observation (*P *= 0.008). The OS rate at 6 years was 92% for R maintenance therapy vs. 86% for observation [35]. Another CD20-targeting immunotherapy—obinutuzumab—is currently being compared with rituximab as maintenance therapy following immunochemotherapy in advanced, CD20-positive indolent FL and MZL patients [36].

In this study, there are several crucial points that require interpretations. The patients’ pathology and imaging, including PET-CT, results were not reviewed centrally. The relatively high rate of treatment discontinuation in this study is a potential caveat. In total, 14 (30%) of the 47 eligible patients enrolled in this study discontinued for reasons such as withdrawn consent, disease progression, AEs, loss to follow-up, or death. Combined with the small target sample size, this high proportion of discontinuations means that conclusions for this study are based on a limited population size. In addition, splenic MZL, which is well-controlled with rituximab monotherapy and maintenance [37], was not included in our study due to it being extremely rare in Korea. Therefore, further study with new novel agents and randomized designed phase III investigating the role of maintenance and induction regimen is needed for improving the survivals of MZL patients.

## Conclusions

In conclusion, this single-arm, open-label, multicenter phase II study of rituximab-maintenance following first-line R–CVP therapy demonstrated good PFS in patients with advanced-stage MZL, with tolerable toxicities.

## Data Availability

The datasets obtained and analyzed during the present study are available from the corresponding author on reasonable request.

## Abbreviations

AEs: adverse events
BM: bone marrow
CI: confidence interval
CISL: Consortium for Improving Survival of Lymphoma
CR: complete response
CT: computed tomography
ECOG PS: Eastern Clinical Oncology Group performance status
FL: follicular lymphoma
HR: hazard ratio
IELSG: International Extranodal Lymphoma Study Group
IWG: International Working Group
LDH: lactate dehydrogenase
MALT: mucosa-associated lymphoid tissue
MZL: marginal zone lymphoma
NCI-CTCAE: National Cancer Institute Common Terminology Criteria for Adverse Events
NHL: non-Hodgkin’s lymphoma
OS: overall survival
PD: progressive disease
PET: positron emission tomography
PFS: progression-free survival
PR: partial response
R–B: rituximab–bendamustine
R–Cb: rituximab–chlorambucil
R–CVP: rituximab, cyclophosphamide, vincristine, and prednisolone
SD: stable disease
TEAEs: treatment-emergent AEs
WHO: World Health Organization
